# Physicochemical Effects of *Lactobacillus plantarum* and *Lactobacillus casei* Cocultures on Soy–Wheat Flour Dough Fermentation

**DOI:** 10.3390/foods9121894

**Published:** 2020-12-18

**Authors:** Bernadette-Emőke Teleky, Gheorghe Adrian Martău, Dan Cristian Vodnar

**Affiliations:** 1 Institute of Life Sciences, University of Agricultural Sciences and Veterinary Medicine, Calea Mănăștur 3-5, 400372 Cluj-Napoca, Romania; bernadette.teleky@usamvcluj.ro (B.-E.T.); adrian.martau@usamvcluj.ro (G.A.M.); 2 Faculty of Food Science and Technology, University of Agricultural Sciences and Veterinary Medicine, Calea Mănăștur 3-5, 400372 Cluj-Napoca, Romania

**Keywords:** dough fermentation, lactic acid bacteria, rheology, soy flour, wheat flour, organic acid

## Abstract

In contemporary food production, an important role is given to the increase in the nutritional quality of foodstuff. In the bakery industry, one of the main cereals used is wheat flour (WF), which creates bread with proper sensory evaluation but is nutritionally poor. Soy-flour (SF) has increased nutrient content, and its consumption is recommended due to several health benefits. Dough fermentation with lactic acid bacteria (LAB) increases bread shelf life, improves flavor, and its nutritional quality, mostly due to its high organic acid production capability. In the present study, the addition of SF to WF, through fermentation with the cocultures of *Lactobacillus plantarum* and *Lactobacillus casei* was analyzed. Three different batches were performed by using WF supplemented with SF, as follows: batch A consisting of 90% WF and 10% SF; batch B—95% WF and 5% SF; batch C—100% WF. The fermentation with these two LABs presented several positive effects, which, together with increased SF content, improved the dough’s rheological and physicochemical characteristics. The dynamic rheological analysis exhibited a more stable elastic-like behavior in doughs supplemented with SF (G′ 4936.2 ± 12.7, and G″ 2338.4 ± 9.1). Organic acid production changes were the most significant, especially for the lactic, citric, and tartaric content.

## 1. Introduction

The global food industry confronts serious challenges to provide consistent nutrition [1,2]. Cereals are classified among the primary food resources worldwide [3], wheat (*Triticum aestivum* L.) represents an essential cereal produced in the world, and it is also highly consumed, especially in Europe [4]. In the bakery industry, doughs are made mainly of refined wheat flour, owing to its particular viscoelastic features resulting mostly from the gluten network (gliadins and glutenins). Although bread produced from wheat flour (WF) has a high energy source, nutritionally, it is unsatisfactory with low mineral and fiber content [5]. Several studies search for alternative ingredients (i.e., amaranth, aleurone, potato, quinoa, etc.) as a substitute, in bakery products to increase its nutritional quality [5,6,7,8]. Soybean (*Glycine max* L.) is part of the Fabaceae family, with particularly high nutrient content like carbohydrates, lipids, proteins, minerals, fibers, vitamins, and has low saturated fat content [9,10]. Soybeans are rich in isoflavones (phytoestrogens) like daidzein and genistein, which specifically contribute to the biological activity of soybean that aroused considerable interest in functional food preparation [11]. As demonstrated, fermentation improves the sensory and physicochemical characteristics of soy-based products and has several health benefits [12].

**Figure 1:**
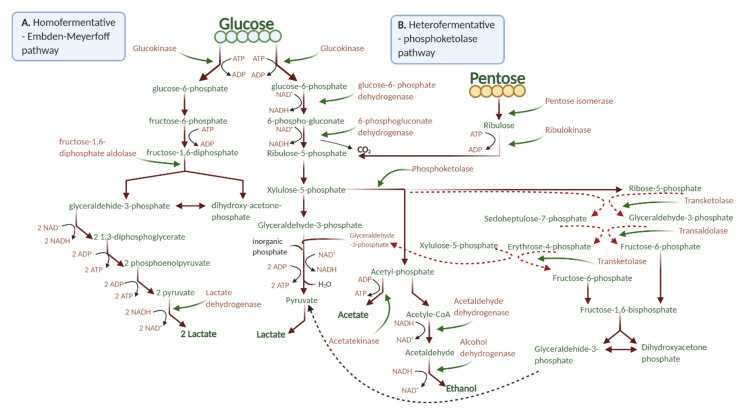
Glucose fermentation pathway. (**A**) Homofermentative metabolism (through Embden–Meyerhoff pathway); (**B**) heterofermentative metabolism (phosphoketolase pathway) (image created using BioRender application https://app.biorender.com).

An ancient biotechnological process is sourdough fermentation that is highly used in the food industry and was a unique bread leavening technique preceding the breakthrough of yeast fermentation [13]. Generally, sourdough fermentation has a positive effect on WF-based products, as a consequence of low pH on dough constituent’s structure formation like arabinoxylans, starch, and gluten. Lactic acid bacteria (LAB) have a positive influence on bread quality, especially regarding shelf life, texture, nutrition, and flavor resulting in a product free of additives [14]. Through fermentation, carbohydrates are consumed being essential for the growth of LAB, and starch metabolization also leads to a drop in pH which enhances organic acid production [15]. LAB also have the ability to metabolize through hydrolysis the nondigestible oligosaccharides found in soy, like glycinin G1, β-conglycinin, and 2S albumin giving the final product flavors and aroma [16,17]. *Lactobacillus plantarum* (*LP*) is a facultative heterofermentative bacteria that use the Embden–Meyerhof–Parnas (EMP) pathway (Figure 1A) to break down glucose and generate lactic acid [18,19,20].

This bacteria adapts easily, has an enhanced sourdough acidification rate, and is pliable with other bacteria or yeasts in cocultures [21]. *Lactobacillus casei* (*LC*) has the same adaptability, like *LP*, and can generate lactic acid through EMP (Figure 1A) and phosphoketolase pathways (Figure 1B), which leads to homo- and heterolactic fermentation [21]. *LC* is widely incorporated in foodstuffs owed to its appealing technological characteristics and health benefits [22,23]. Furthermore, dough fermentation has several beneficial effects, like the pretreatment of the substrate, through increasing and stabilizing the functional quality of the fermented dough. Moreover, it produces a diminished glycemic reaction, positively impacts allergic and intolerance effects in individuals, and increases the absorption of vitamins, minerals, and phytochemicals [24,25].

Through fermentation the rheological property of dough is influenced by many factors, like the content of sodium [26], the type of microorganism found in the fermented dough [16,27], the type of substrate used [14,28], and by the addition of different oleogels [29], proteins [30,31,32], or alternative sweeteners [33,34]. The impact of soy proteins on the wheat dough is highly studied [35,36]. The viscoelastic characteristics of wheat dough are given by its gluten network, and the consequence of incorporating different proteins might have a negative impact on bread properties [37]. The incorporation of soy-flour (SF) presents foam-stabilizing, moisture-holding potential, gelation, emulsifying activities, and through disulphide-linkages offers elasticity to bread products [32]. Additionally, a recent study proved that the enzyme β-glucosidase found in LAB can effectively increase the aglycone content of soybean products. An important aspect that should be analyzed through bread making is the property of dough after frozen storage, which can be effectively characterized through rheology [38].

The cocultivation of two LAB (*LP* and *LC*) on WF-SF substrates has not been studied only together with *Saccharomyces cerevisiae* (*Sc*) and especially on model media (MM) [21,39]. Fermentation with only LAB cocultures is important to see their competitiveness and organic acid production capabilities in bakery products. Additionally, the use of LAB as starter cultures in dough fermentation can increase the mineral availability, due to phytate degrading enzymes [40]. Only a few studies analyzed dough fermented exclusively by LAB, which could provide help to people suffering from various health problems [41]. An important aspect that needs to be better analyzed is the change during fermentation with LAB on different substrates and the effect of SF incorporation in WF doughs. Therefore, the present article has the objective to investigate the impact of SF incorporation in WF, through LAB coculture fermentation with *LP* and *LC*. Besides the dynamic rheological properties of three different concentrations of WF and SF doughs, the chemical properties, cell growth, and pH were also evaluated.

## 2. Materials and Methods

### 2.1. Strains and Culture Conditions

The fermentations involved two types of LAB, namely *Lb. plantarum* ATCC 8014 (*LP*) and *Lb. casei* ATCC 393 (*LC*) acquired from the University of Agricultural Science and Veterinary Medicine Cluj-Napoca. Culture media constituent elements and every reagent were of analytical grade. For both microorganisms MRS broth (casein peptone—tryptic digest 10 g/L; meat extract 10 g/L; yeast extract 5 g/L; glucose 20 g/L; Tween 80 1 g/L; K_2_HPO_4_ 2 g/L; Na-acetate 5 g/L; (NH_4_)_3_ citrate 2 g/L; MgSO_4_ × 7 H_2_O 0.2 g/L; MnSO_4_ × H_2_O 2 g/L; distilled water 1000 mL) was used.

### 2.2. Dough Preparation

The soy-flour utilized in this study originated from the Agricultural Research and Development Center Turda. The soybean type was Onix (*Glycine max* (L.) Merril), with a traditional cultivation method (tillage) using 60% of green fertilizer (vegetable debris). A commercially available wheat flour (type 000, in conformity with Romanian ash content categorization) was used, having 11.2% protein and 15.3% moisture content. Three types of flour concentrations were prepared for fermentation, composed of 90% WF enriched with 10% SF (batch A), 95% WF enriched with 5% SF (batch B), and 100% WF (batch C). The quantity of water added to the different flour concentrations (100 g of flour) was 60% of the whole formulations, while 40% was the inoculum composed of the two LABs used as coculture, with a total dough amount of 200 g. Before inoculation, the different WF-SF concentrations were sterilized, together with the necessary amount of distilled water, after which they were mixed thoroughly and inoculated with the activated and necessary amount of LAB as presented in our previous study Teleky et al. (2020) [39].

### 2.3. Model Media and Dough Fermentation

Model media (MM) cultured the same way as presented in a previous study [39], and the dough was inoculated with the microorganisms *LP* and *LC* in a concentration of 10^8^ CFU/mL. The final volume of MM was 500 mL, and each dough combination had a final volume of 200 mL and was inoculated with the same amount of inoculum from both LAB. Sample extraction occurred at every 4 h (0, 4, 8, 12, 24 h) with 5 mL of sample for high-performance liquid chromatography (HPLC) analysis, 5 mL for pH, 1 mL for viability, and 5 mL for rheological measurements.

### 2.4. Testing Methods

#### 2.4.1. pH

pH evaluation was established with a digital pH meter (InoLab 7110, Wellheim, Germany). Samples were dissolved with 45 mL of distilled water at room temperature and measured while the samples were homogenized continuously with a magnetic stirrer [42].

#### 2.4.2. Cell Viability

LAB viability was established by diluting 1 mL of prelevated sample in 9 mL of sterile saline solution and processed with the spread plate method and incubated for 24 h at 37 °C [43]. The viability of LAB was evaluated through plate counting and colony-forming units/mL sample and was displayed in logarithmic values of colony-forming units/milliliter of the sample (log10 CFU/mL).

#### 2.4.3. Rheological Properties

Samples for rheological analysis were stored at −20 °C, defrosted at room temperature after which they were analyzed with an Anton Paar MCR 72 rheometer (Anton Paar, Graz, Austria) [44,45]. The dynamic rheological measurements were performed with a Peltier plate-plate system (P-PTD 200/Air) supplied with temperature control, and with a smooth parallel plate geometry (PP-50-67300) of 50 mm. Shear strain (oscillating) was set at a constant value of 0.1% and angular frequency (ω) at a logarithmic ramp and set between the intervals of 0.628–628 rad/s. After the sample supplying, the gap between plates was set at 1 mm, the dough surplus was trimmed, and to prevent the sample from drying, silicone oil was distributed on the exterior.

#### 2.4.4. HPLC

Organic acid consumption and production were measured by HPLC (Agilent 1200 series, Santa Clara, CA, USA) after the samples were filtered (for MM) or homogenized (for dough, 1 g of sample with 2 mL of distilled water), vortexed, sonicated, centrifuged, and filtered (0.45 µm pore size). The HPLC was provided with a solvent degasser, DAD detector coupled with a mass detector, quaternary pump, thermostat column, and an automatic injector (Agilent Technologies, Santa Clara, CA, USA). In the column, a volume of 20 µL of the sample was injected, with a 0.5 mL/min sample flow rate, and detection was carried out at 280 and 340 nm. The organic acid separation was accomplished on a reversed-phase chromatographic column Acclaim OA (5 µm, 4 × 150 mm Dionex), washed with NaH2PO4 50 mM concentration (pH 2.8) solution for 10 min, at 20 °C, and with a 0.5 mL/min flow rate. Chromatogram measurement was performed at a λ = 210 nm wavelength [46,47]. Organic acid standard stock solution (Merck) was prepared by mixing the components (lactic, tartaric, malic, citric, succinic, fumaric, butyric acids) (Supplementary Figure S1).

#### 2.4.5. Statistical Analysis

Every measurement was performed in triplicate and expressed as mean value (±SD, n = 3). The statistical interpretation was performed with the help of Graph Prism Version 8.0.1. (GraphPad Software Inc., San Diego, CA, USA) with a one-way ANOVA test (Tukey multiple comparisons tests) [48]. Statistically significant differences of means were considered at a 5% level.

## 3. Results and Discussion

### 3.1. LAB Viability and pH

**Figure 2:**
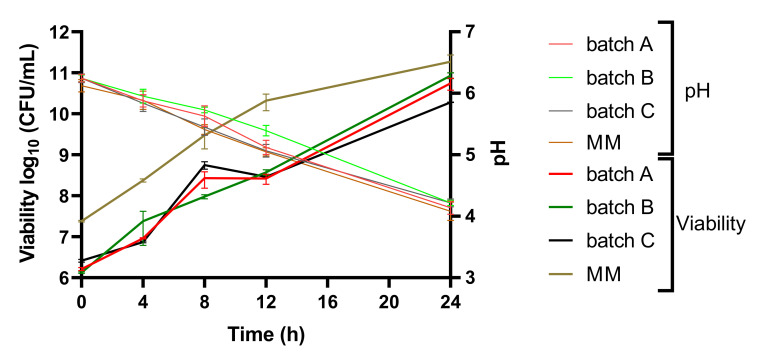
Cell viability (spread plate method) and pH profile of the fermentations at the three different substrate concentrations (wheat and soy-flour) and MM for 24 h with the cocultures. Values for LAB viable cell growth and pH are displayed as mean values ± SD, log10 CFU/mL, n = 3, GraphPad Prism Version 8.0.1 (Graph Pad Software, Inc., San Diego, CA, USA); batch A (90% WF + 10% SF addition); batch B (95% WF + 5% SF addition); batch C (100% WF), MM (model media); CFU/mL (colony-forming units/milliliter of sample).

LABs are one of the most frequently used microorganisms in sourdough fermentation, and the use of appropriate starter cultures have great importance. In SF the carbon:nitrogen ratio is not satisfactory for the growth of LAB, and various carbon sources can influence the metabolism and growth of *LP* and *LC* [49]. Through fermentation on every WF and SF concentration, the growth of LAB cocultures increased from an average value of 6.1–6.4 log10 CFU/mL, which was very similar in every batch, to 10.8–10.9 log10 CFU/mL (Figure 2).

Through the fermentation, the values gradually increased and reached a final concentration of 10.8–10.9 log10 CFU/mL on substrates where SF was added and 10.3 log10 CFU/mL where only WF was the sole substrate. In MM, the viability was considerably higher, with final values reaching 11.5 log10 CFU/mL. The same trend could be observed at viability with the cocultures of *LP*, *LC*, and *Sc* [39], where a final concentration of 10.4 log10 CFU/mL was reached in the case of flour as substrate and 12.5 log10 CFU/mL in the case of MM. Dough enriched with SF had a beneficial effect on viability which can be justified by the ability of LAB to degrade soy as substrate, particularly *LP* [50].

Throughout fermentation, the pH gradually decreased, but in the case of MM and batch C, the pH decreased more rapidly, owing to better accessibility of carbohydrates. A similar decline in pH could be observed in a study on different carbohydrates with starting values of about 6.1 and final values between 4.4 and 4.8 [21]. The decrease in pH is caused by the production of organic acids through fermentation by LAB especially lactic acid. With the continuous metabolic activities of the LAB, the pH reached lower values than <4.3 at the end of fermentation, which can also be seen in several studies led on different substrates [51,52,53].

### 3.2. Viscoelastic Behavior of Dough

Dough rheology became a field with a great deal of interest in the cereal industry, considering its implication in baked product quality [54]. An essential qualitative parameter of bread products is associated with dough’s viscoelasticity, which is given by the gluten complex found in wheat. Through bread-production dough experiences a variety of strains and stresses of different degrees, which are essential in evaluating the viscoelastic behavior of dough. Particularly through fermentation, a significant element is extensional deformation, which has a major impact on the rheological characteristics and consistency of the end-product [32,54].

**Figure 3:**
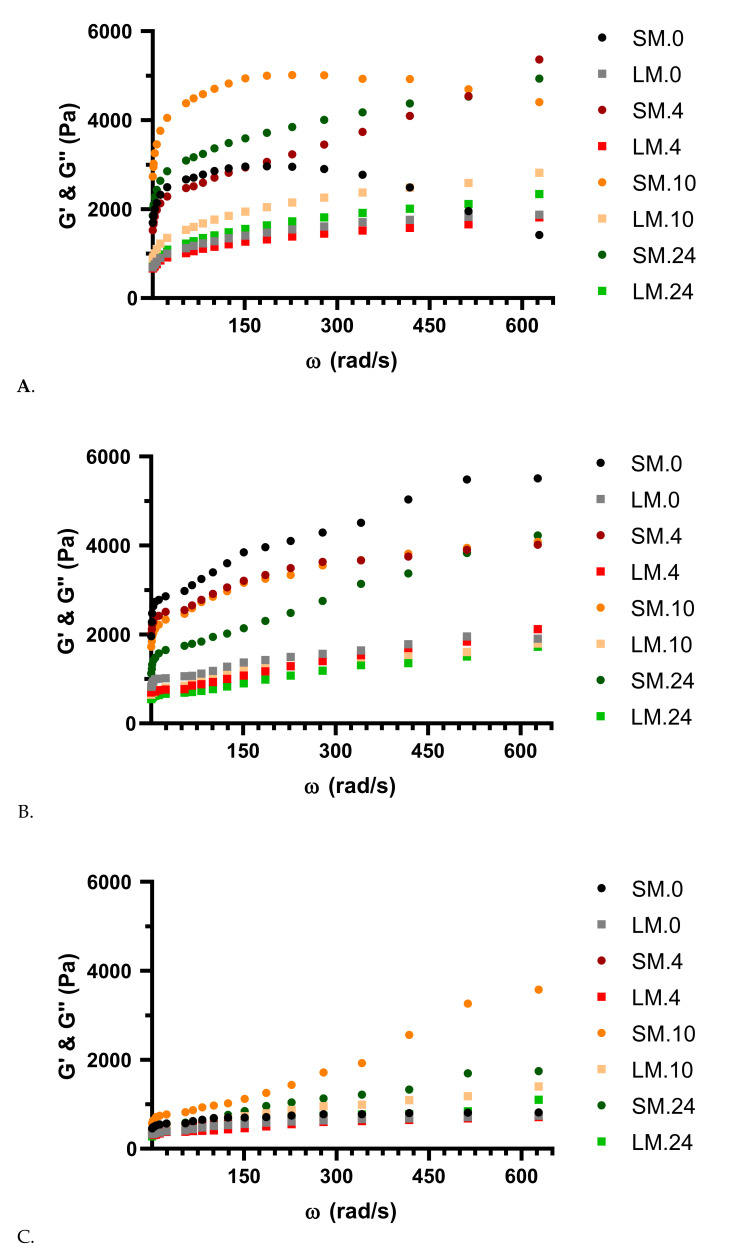
Storage (SM—G′, filled dots ●) and loss modulus (LM—G″, filled squares ▪) performed as a function of angular frequency (ω) in doughs at different strengths for (**A**) batch A (90% WF + 10% SF addition); (**B**) batch B (95% WF + 5% SF addition); (**C**) batch C (100% WF) fermented with the coculture of *LP* and *LC* through a 24 h period.

In Figure 3, and Supplementary Tables S1–S3, the viscoelastic properties of doughs at 30 °C in all three wheat–soy flour concentrations (batches A, B, and C) through coculture fermentation with *LP* and *LC* are presented. Every dough combination showed with the increase of angular frequency (ω) a rise in storage (G′) and loss modulus (G″).

The moisture content of mixtures of WF and SF doughs have different water absorption capacities, which affects the dynamic moduli (with lower water content it amplifies) [55]. As a consequence, the water content of every batch was the same in every experiment. Considering that in every dough sample, the G′ was higher than the G″, and also increased with the increase of ω. Consequently, every dough sample presented a weak gel-like behavior (stable elastic behavior). The highest gel-like behavior was observed in the batches where SF was added, with final values at 24 h fermentation of G′ 4936.2 ± 12.7, and G″ 2338.4 ± 9.1 for batch A and G′ 4225.2 ± 11.9, and G″ 1726.7 ± 10.1 for batch B. The same behavior was reported in several studies where different types of flours were analyzed in dough production, like aleurone, durum, Psyllium, amaranth flour, corn starch, and pea isolate [5,7,56].

Although the addition of SF to WF has a negative impact on bread volume, the sour flavor given by the organic acids hides the odd flavor of soybean, and as a result, the consumer acceptance is favorable [57]. Similar reports stated that soy proteins, besides giving valuable nutritional quality to doughs, also display multiple functional characteristics like emulsifying, gelation action, moisture retention capability, and stabilize the foam activity, which has a good influence on dough products [32,36]. Furthermore, dough fermentation influenced the rheological property of wheat/wheat–soy doughs becoming flexible, easily extensible, with low elasticity [13]. In the batch where no SF was added, G′, and G″ had both very low values, although in these samples a small elastic behavior was also observed, with SF addition, this behavior was accentuated.

### 3.3. Organic Acid Production

Sourdough fermentation is an ancient bread-making technique, required for dough leavening. Through fermentation, LAB, which usually exists in flour, produces lactic acid. The production of an appropriate quantity of volatile compounds needs fermentation of approximately 12–24 h, whereas, with baker’s yeast, it is sufficient for a couple of hours. For the acidification of dough, usually, the LAB is accountable. Lactic acid and acetic acid play an essential role in the general flavor perception of the produced bakery products. In the production of organic acids, an important role is given to the fermentation temperature, the utilized flour type, and especially the used starter culture (homo- or heterofermentative LAB) [58]. Through EMP, LAB ferment glucose, and carbohydrates are conveyed by the phosphotransferase system transporter systems. Besides glucose, sugars are metabolized under carbon-catabolite regression, fructose is entirely utilized as a carbon source, and pyruvate acts as the main connection point of metabolism [59].

**Figure 4:**
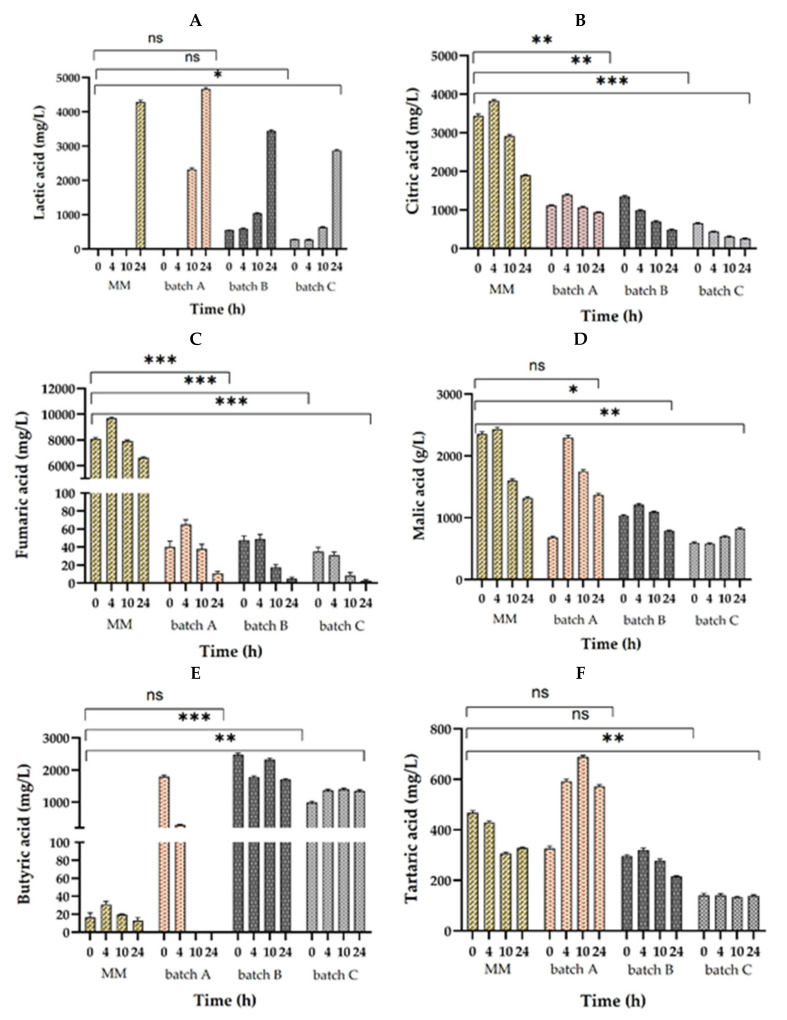
Organic acid production through 24 h fermentation with the cocultures *LP* and *LC* in MM (model media—control); batch A (90% WF and 10% SF), B (95% WF and 5% SF), and C (100% WF); (**A**) lactic acid, (**B**) citric acid, (**C**) fumaric acid, (**D**) malic acid, (**E**) butyric acid, and (**F**) tartaric acid. Values displayed as mean ± SD of the triple measurements and symbols (ns, *, **, ***) express the not-significant or significant differences (*p* < 0.05) between the MM and the three batches (one-way analysis of variance (ANOVA), multiple comparison tests).

Considering lactic acid production (Figure 4A), at the beginning of fermentation it was either elevated or with no production at all, after which the highest level was observed in batch A with 4661.1 mg/L, followed by batch B with 3440.9 mg/L and the lowest quantity was 2872.9 mg/L in batch C. This organic acid was found in a higher amount of 12,306.3 mg/L in a similar study, on amaranth flour dough fermentation with three LAB strains, *LP* RTa12, *Lb. sakei* RTa14, and *Pediococcus pentosaceus* RTa11 [60]. On different semolina and pistachio powder enriched WF sourdoughs with mixed LAB cocultures, lactic acid was produced between 5210 and 5890 mg/L without pistachio powder, and 7470–9800 mg/L in enriched flours, which also resulted in bread with better sensory analysis [53].

Heterofermentative LAB in the fermentation process achieves acidification by organic acid production, especially by lactic and acetic acids, which are also accountable for bread products’ shelf-life extension [61]. Even though acetic acid production in the fermented dough was not observed in any of the three batches, in MM from an initial quantity of 3437.7 mg/L at 0 h, it decreased to 1892.3 mg/L at 24 h which is in accordance with similar studies [21,62].

Alongside lactic acid and acetic acid, several organic acids have antimicrobial activities, like citric acid, fumaric acid, malic acid (Figure 4D), tartaric acid (Figure 4F), and butyric acid. Through the decarboxylation of malic acid, lactic acid is produced [63], which in our studies decreased to values of 1366.107 (batch A), 786.589 (batch B), and 824.863 mg/L (batch C).

In the three wheat–soy flour doughs, citric acid (Figure 4B) presented inconstant results through fermentation, because generally it is consummated when LAB are low in carbohydrates. Still, at 24 h, the quantity decreased to 931.7, 479.6, and 256.9 mg/L in the three batches. Simurina et al. (2014) stated that the incorporation of citric acid substantially enhances bread quality, and has crumb softening aftermath [64]. Fumaric acid (Figure 4C), although recognized as an advantageous organic acid on human health as a result of its antioxidant activity [65], in these batches it was found in a low quantity. In the three batches from the beginning of the fermentation, it decreased from 35.051–47.510 to 2.673–10.835 mg/L at 24 h. Su et al. (2019) found that organic acids (lactic, citric, acetic, malic, and fumaric) have a good effect on specific volume, decrease the moisture content, pH, and bread hardness. On the other hand, organic acids also decreased the gas retention capacity and diminished the gluten network [65].

Butyric acid (Figure 4E) is especially beneficial for colon health and has the added capacity to prevent or to treat Crohn’s disease, cancer, and distal ulcerative colitis [66]. The production of butyric acid decreased till the end of fermentation, reaching values of 1711.167 mg/L in batch B and 1349.320 mg/L in batch C. In batch A, the butyric acid quantity was not detected after 24 h of fermentation.

The highest concentration of organic acids (except butyric acid) at the end of fermentation was found in batch A, which indicates that SF addition improves considerably organic acid production. With a final concentration of tartaric acid of 572.508 mg/L, malic acid of 1366.107 mg/L, lactic acid of 4661.098 mg/L, citric acid of 931.690 mg/L, and fumaric acid of 10.835 mg/L, the addition of 10% of SF has an improved effect on dough quality. In comparison with our earlier results with single fermentation of *LP*, *LC*, or coculture fermentation of *LP* + *LC* + *Sc* [39], the coculture of *LP* + *LC* without yeast had the highest organic acid production. *Sc* generates high quantity of CO_2_ which is accountable for dough leavening. LAB are also accelerated by CO_2_, and inhibited by the aerobically produced reactive oxygen species [67]. The lower lactic acid concentration can be explained by the fact that *Sc* consumes this organic acid from dough, but this way [68] increases the growth of LAB by reducing the acidification of the substrate [68].

Cocultivation of *LP* and *LC* led to a high concentration of lactic acid, especially where SF was present. As in our earlier study carbohydrate metabolization was better with *LP* than with *LC*, especially in lactic acid production. In general, in single cultures, organic acid production was higher with *LP* which indicates that it has a dominant trait in carbohydrate consumption. However, on every substrate combination both LAB showed efficient synergy.

## 4. Conclusions

Dough fermentation with cocultures of *LP* + *LC* presented favorable results in organic acid development, especially with an increased lactic (4661.098 mg/L), citric (931.690 mg/L), and tartaric (572.508 mg/L) acid production. The viability of these two LABs, grown at 37 °C, increased to 10.28–11.51 log10 CFU/mL, and the pH decreased at values between the range of 4.14–4.23. These two LAB had good metabolite production efficiency and both *Lb.* strains were in symbiosis with favorable growth dynamics.

In the course of fermentation dough experiences important rheological alterations, which were investigated using dynamic rheological measurements. Besides, the effect of SF incorporation (up to 10%) in WF produced several positive changes in the rheological characteristics of dough, like increased elastic-like behavior. Additionally, with the increase of SF, an increase in organic acid content could be observed, which further supports the addition of SF in bakery products.

Further research should be run to evaluate the positive effects of other microorganisms like (i.e., *Lb. florum*, *Lb. bulgaricus*, *Oenococcus oeni*, etc.) through dough fermentation in single or cocultures, together or without the addition of baker’s yeast (*S. cerevisiae*), and also the effect of higher substitution of SF to WF. As a future scope, the consequence of SF addition to WF through frozen storage in comparison with the unfrozen dough will be performed. The interferences of LAB in cocultures and their metabolic activity with the effect on dough characteristics is important.

## Supplementary Materials

The following are available online at https://www.mdpi.com/2304-8158/9/12/1894/s1, Figure S1. HPLC chromatogram of organic acid standards and retention times; Table S1. Cell viability and pH profile of the fermentations at the three different substrate concentrations (wheat and soy-flour) and MM for 24 h with the cocultures; Table S2. The standard deviation of storage (G′) and loss (G″) shear moduli for 100% wheat flour with *LP* + *LC* cocultures; Table S3. The standard deviation of storage (G′) and loss (G″) shear moduli for 95% wheat flour and 5% soy flour with *LP* + *LC* cocultures; Table S4. The standard deviation of storage (G′) and loss (G″) shear moduli for 90% wheat flour and 10% soy flour with *LP* + *LC* cocultures.
