# Supplementary material for: Physicochemical Effects of Lactobacillus plantarum and Lactobacillus casei Cocultures on Soy–Wheat Flour Dough Fermentation
Source: Foods. 2020 Dec 18;9(12):1894. doi: 10.3390/foods9121894 (PMC7766497; doi:10.3390/foods9121894)
Supplement: Supplementary file 1 [file foods-09-01894-s001.pdf]

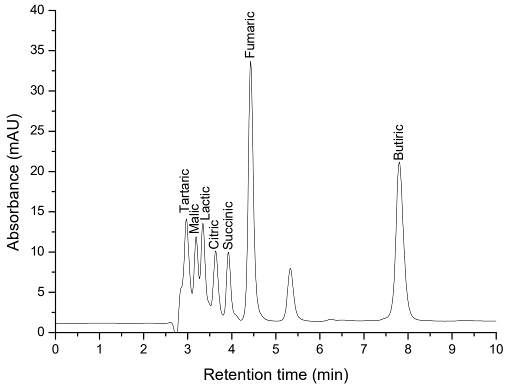

Retention time (min)	Organic acid
2.97	Tartaric
3.18	Malic
3.34	Lactic
3.65	Citric
3.97	Succinic
4.42	Fumaric
7.80	Butyric

Supplementary Figure 1. HPLC chromatogram of organic acids standards and retention times.

Supplementary Table S1. Cell viability and pH profile of the fermentations at the three different substrate concentrations (wheat and soy-flour) and MM for 24 h with the co-cultures

	**Time (h)**	**0**	**4**	**8**	**12**	**24**
**MM**	**pH**	6.12 ± 0.07	5.95 ± 0.08	5.40 ± 0.11	5.13 ± 0.07	4.06 ± 0.08
	**viability**	7.40 ± 0.33	8.01 ± 0.27	9.30 ± 0.24	10.39 ± 0.31	11.51 ± 0.47
**Batch A**	**pH**	6.24 ± 0.03	5.97 ± 0.05	5.66 ± 0.14	5.11 ± 0.02	4.14 ± 0.03
	**viability**	6.20 ± 0.26	7.01 ± 0.31	8.30 ± 0.35	8.34 ± 0.28	10.77 ± 0.45
**Batch B**	**pH**	6.24 ± 0.06	5.91 ± 0.11	5.74 ± 0.05	5.41 ± 0.09	4.23 ± 0.04
	**viability**	6.11 ± 0.13	6.42 ± 0.35	7.98 ± 0.26	8.50 ± 0.21	10.99 ± 0.37
**Batch C**	**pH**	6.25 ± 0.07	5.82 ± 0.14	5.41 ± 0.14	5.07 ± 0.11	4.22 ± 0.07
	**viability**	6.42 ± 0.19	6.87 ± 0.23	8.08 ± 0.28	8.46 ± 0.24	10.28 ± 0.41

Results (displayed as mean values ±SD, n = 3), MM – model media; batch A - 90% WF enriched with 10% SF; batch B - 95% WF enriched with 5% SF; batch C - 100% WF.

Supplementary Table S2. Standard deviation of storage (G′) and loss (G′′) shear moduli for 100% wheat flour with *LP* + *LC* co-cultures.

[rad/s]	SM.0	LM.0	SM.4	LM.4	SM.10	LM.10	SM.24	LM.24
**0.943**	455.73 ± 3.6	334.53 ± 4.6	439.19 ± 5.1	338.41 ± 3.3	572.67 ± 4.8	383.66 ± 5.1	381.33 ± 8.5	275.23 ± 4.1
**1.73**	481.85 ± 11	329.28 ± 7.2	426.23 ± 8.5	321.23 ± 5	606.1 ± 8.6	386.07 ± 4.4	450.05 ± 11	337.92 ± 4.6
**2.13**	484.07 ± 9.7	336.42 ± 5.6	466.37 ± 7.1	321.78 ± 2.8	630.62 ± 2.9	390.29 ± 1.7	461.89 ±5.6	338.05 ± 3.9
**3.91**	497.02 ± 12	339.03 ±4.3	467.86 ± 2.1	308.7 ± 9	649.06 ± 8.5	394.74 ± 3.9	484.22 ± 6.8	365.68 ± 6.8
**7.19**	521.71 ± 8.5	355.06 ± 8.4	510.92 ± 6.2	318.09 ± 1.8	710.96 ± 4.2	412.32 ± 2.8	509.26 ± 9.1	394.28 ± 5.5
**13.2**	546.24 ± 7.2	362.44 ± 6.6	522.44 ± 9.5	343.23 ± 8.5	743.5 ± 1.1	426.47 ± 7.5	523.24 ± 6.4	442.01 ± 10
**24.3**	564.6 ± 10.1	394.06 ± 5.8	568.28 ± 11	374.83 ± 3.3	769.93 ± 9.7	471.37 ± 11	555.6 ± 5.7	435.52 ± 11.1
**54.8**	571.57 ± 9.3	411.75 ± 9.1	566.03 ± 5.6	381.5 ± 6.8	822.22 ± 6.1	496.83 ± 2.3	585.65 ± 3.8	461.33 ± 8.9
**67.2**	623.09 ± 8.8	444.03 ± 11	582.56 ± 7.1	395.84 ± 2.3	868.43 ± 11	532.36 ± 5.2	594.5 ± 4.1	490.58 ± 7.1
**82.3**	653.68 ± 10	476.94 ± 7.3	600.05 ± 5.2	401.28 ± 4.3	928.59 ±8.5	577.68 ± 7.1	608.17 ± 5.8	538.53 ± 4.9
**101**	687.55 ± 9.4	510.23 ± 7.7	592.38 ± 9.8	410.34 ± 5.2	973.16 ± 9.9	613.36 ± 9	691.85 ± 6.7	564.81 ± 6.1
**124**	701.91 ± 5.5	534.07 ± 6.8	624.91 ± 5.4	434.38 ± 9.5	1021.4 ± 3.6	678.67 ± 7.6	761.18 ± 3.5	601.12 ± 10
**151**	702.76 ± 6.4	556.42 ± 6.4	651.26 ±6.3	462.61 ± 5.8	1121.8 ± 1.9	729 ± 11.3	846.64 ± 2.2	644.9 ± 13.4
**186**	713.51 ± 7.1	586.63 ± 7.1	674.34 ± 8.3	502.17 ± 8	1256 ± 4.1	797.55 ± 3.4	959.31 ± 3.8	697.17 ± 5.6
**227**	747.71 ± 12	613.76 ± 8.2	695.19 ± 9	551.57 ± 5.2	1435.6 ± 9.5	869.51 ± 6.7	1041.9 ± 4.9	725.08 ± 2.8
**279**	777.95 ± 9.9	634.3 ± 4.1	750.98 ± 1.2	599.03 ± 5.6	1715.8 ± 6.7	958.14 ± 5.5	1130.6 ± 6.7	742.41 ± 8.9
**341**	778.08 ± 14	665.96 ± 12	773.98 ± 4.1	620.61 ± 8.9	1924.1 ± 7.4	992.64 ± 2.4	1214.5 ± 3.2	771.98 ± 1.9
**418**	802.52 ± 8.7	679.9 ± 9.1	781.95 ± 5.7	648.57 ±4.4	2557.7 ± 1.2	1089.9 ± 1.7	1332.4 ± 12	774.44 ± 5.1
**513**	811.12 ± 3.4	711.34 ± 1.7	779.87 ± 8.1	678.89 ± 3.2	3259.6 ± 8.9	1178.1 ± 6.7	1696.3 ± 7.8	843.59 ± 6.8
**628**	816.62 ± 11	742.19 ± 5.9	790 ± 12.2	709.63 ± 11	3578.6 ± 7.1	1398.6 ± 8.2	1743.4 ± 9.3	1102.4 ± 7.1

Results (displayed as mean values ±SD, n = 3), SM – storage modulus, LM – loss modulus.

Supplementary Table S3. Standard deviation of storage (G′) and loss (G′′) shear moduli for 95% wheat flour and 5% soy flour with *LP* + *LC* co-cultures.

[rad/s]	**SM.0**	**LM.0**	**SM.4**	**LM.4**	**SM.10**	**LM.10**	**SM.24**	**LM.24**
**0.943**	1957.3 ± 9.9	821.35 ± 7.4	2014.2 ± 7.1	695.37 ± 8.1	1723.4 ± 3.5	645.69 ± 1.9	1126.5 ± 5.6	544.71 ± 13
**1.73**	2281 ± 8.3	841.82 ± 5.9	2120.8 ± 1.4	711.04 ± 4.6	1850.9 ± 8.2	650.08 ± 5.7	1227.3 ±7.7	551.26 ± 5.6
**2.13**	2473.5 ± 7.4	899.06 ± 6.1	2213.9 ± 5.6	708.14 ± 3.1	1956.4 ± 2.6	648.03 ± 12.1	1319.8 ± 8.7	572.62 ± 3.7
**3.91**	2634.6 ± 11	944.89 ± 3.7	2278.7 ± 3.6	714.92 ± 9.4	2028.4 ± 4.7	696.45 ± 6.7	1413.6 ± 9.8	599.3 ± 6.8
**7.19**	2731.9 ± 5.6	996.57 ± 9.4	2394.5 ± 5.2	717.2 ± 11.6	2123.2 ± 5.6	740.62 ± 8.1	1476.5 ±14	619.64 ± 9.9
**13.2**	2777.3 ± 4.3	1006.7 ± 7.5	2418.4 ± 5	746.26 ± 7.9	2217.2 ± 3.7	756.42 ± 9.8	1575.2 ± 5.6	636.56 ±3.8
**24.3**	2859.4 ± 8.1	1016.1 ± 4.4	2507.1 ± 7.5	758.98 ± 6.8	2332.6 ± 5.5	807.5 ± 2.8	1650.5 ± 6.7	666.52 ± 3.7
**54.8**	2979 ± 7.9	1059.8 ± 9.2	2550 ± 7.2	773.56 ± 4.7	2462.8 ± 10	844.23 ± 4.4	1745.3 ± 7.4	692.26 ± 1.9
**67.2**	3106.8 ± 10	1068.9 ± 2.5	2653.1 ± 1.9	850 ± 12.3	2587 ± 9.1	892.4 ± 5.6	1791.2 ± 6.3	707.73 ± 3.6
**82.3**	3247.2 ± 1.3	1121.8 ± 5.1	2779.1 ± 5.1	879.54 ± 8.7	2725.6 ± 6.7	943.08 ± 7.4	1839.3 ± 3.7	733.24 ± 7.1
**101**	3397.9 ± 5.6	1181.2 ± 7.9	2914 ± 4.1	932.66 ± 3.9	2848.7 ± 1.9	1005.1 ±6.5	1941.9 ± 6.6	773.45 ± 6.5
**124**	3602.4 ± 6.8	1272.9 ± 4.2	3056.6 ± 4.2	999.38 ± 9.1	2974.1 ± 2.6	1102.6 ± 3.7	2016.8 ± 9	832.18 ± 5.1
**151**	3847.3 ± 7.8	1372.3 ± 7.5	3206.8 ± 8.8	1078.2 ± 5.3	3164.2 ± 7.4	1190.8 ± 4.1	2141.2 ± 7.2	901.74 ± 3.7
**186**	3962.3 ± 5.5	1426.4 ± 2.7	3338 ± 2.7	1172.7 ± 5.9	3249.7 ± 8.7	1241.2 ±3.7	2305.5 ± 5.9	985.19 ± 8.1
**227**	4103 ± 7.8	1496.1 ± 8.7	3493.9 ± 8.4	1284 ± 6.7	3337.1 ± 3.6	1297.5 ±7.8	2482.7 ± 6.4	1073.3 ± 1.9
**279**	4288.7 ±8.4	1563.9 ± 1.2	3632.9 ± 3.8	1398 ± 8.8	3551.8 ± 6.4	1418.8 ± 2.4	2752.2 ± **3.4**	1187.2 ± 3.3
**341**	4508.7 ± 4.1	1640.1 ± 11	3663.9 ± 2.4	1537 ± 5.9	3671.2 ± 5.2	1486 ± 9.7	3136.4 ± 5.2	1308.4 ± 7.6
**418**	5033.9 ± 8.7	1779.9 ± 10.3	3749.1 ± 3.9	1692.4 ± 6	3814.4 ± 5.4	1540 ± 3.4	3372.3 ± 6.2	1353.6 ± 8.4
**513**	5483.4 ± 8.5	1953.3 ± 8.6	3898.6 ± 2.5	1840 ± 5.7	3944.4 ± 2.2	1603.9 ± 6.6	3830.8 ± 9.7	1503.9 ± 2.4
**628**	5509.4 ± 11.1	1905.9 ± 3.6	4017.4 ± 6.7	2121 ± 8.9	4079.7 ± 3.7	1792.4 ± 10	4225.2 ± 9.4	1726.7 ± 7.8

Results (displayed as mean values ±SD, n = 3), SM – storage modulus, LM – loss modulus.

Supplementary Table S4. Standard deviation of storage (G′) and loss (G′′) shear moduli for 90% wheat flour and 10% soy flour with *LP* + *LC* co-cultures.

[rad/s]	**SM.0**	**LM.0**	**SM.4**	**LM.4**	**SM.10**	**LM.10**	**SM.24**	**LM.24**
**0.943**	1695 ± 1.8	691.2 ± 6.4	1522.6 ± 8.9	665.24 ± 2.9	2734 ± 7.2	891.39 ± 5.6	1853.9 ± 11	725.28 ± 7.7
**1.73**	1837.8 ± 7.4	707.1 ± 3.4	1661.9 ± 2.2	679.64 ± 5	2946.8 ± 7.7	931.88 ± 4.7	2046.8 ± 8.4	754.62 ± 8.4
**2.13**	1876.2 ± 13	715.4± 4.7	1705.1 ± 3.8	692.37 ± 3.5	3021.3 ± 13.1	941.34 ± 1.4	2106.3 ± 7.6	763.94 ± 10.6
**3.91**	2001.4 ± 2.6	758.6 ± 5.6	1834.6 ± 10.8	722.6 ± 8.8	3256.4 ± 10.7	1006.8 ± 5.4	2268.4 ± 2.8	817.31 ± 3.1
**7.19**	2141 ± 5.9	815.77 ± 11	1977.1 ± 7.1	763.36 ± 5.4	3458.1 ± 4.1	1103.7 ± 8.4	2432.8 ± 5.3	888.97 ± 13.5
**13.2**	2321.3 ± 6.9	899.93 ± 7.9	2129 ± 4.9	843.83 ± 7.4	3763.1 ± 7.2	1224.2 ± 7.1	2639.4 ± 5.9	980.64 ± 5.6
**24.3**	2495.7 ± 4.6	1000 ± 8.9	2286.1 ± 3.5	916.4 ± 4.8	4052 ± 7.9	1357.4 ± 9.4	2853 ± 14.1	1093.1 ± 6.7
**54.8**	2670.2 ± 5.9	1118.5 ± 6.4	2473.4 ± 4.4	1012 ± 8.5	4377.9 ± 3.1	1534.5 ± 5.7	3090.1 ± 6.8	1226.7 ± 8.7
**67.2**	2707.9 ± 9.2	1176.6 ± 5.4	2511.4 ± 9.3	1057.2 ± 4.4	4490.9 ± 9.8	1598 ± 3.7	3167.7 ± 6.5	1280.5 ± 3.9
**82.3**	2778.5 ± 4.7	1230.4 ± 3.1	2590.7 ± 6.8	1109.2 ± 3.1	4585.5 ± 15	1679.9 ± 8.1	3244.4 ± 5.3	1348.7 ± 4.5
**101**	2859.2 ± 3	1282.1 ± 9.8	2706.9 ± 6	1153.2 ± 8.4	4705.4 ± 9.7	1764.4 ± 5.9	3369.3 ± 1.5	1411.7 ± 6.1
**124**	2919 ± 12.4	1343.2 ± 6.1	2815.8 ± 5.1	1209.3 ± 2.4	4824.8 ± 6.4	1849.9 ± 4.9	3486.5 ± 4.3	1480.3 ± 7.4
**151**	2959.8 ± 5.6	1407 ± 5.3	2936.3 ± 9.3	1268.6 ± 5.6	4938.7 ± 4.1	1943.4 ± 3.7	3593.3 ± 4.8	1554.4 ± 8.9
**186**	2961.5 ± 7.1	1473.9 ± 6.1	3063.8 ± 6.9	1318.8 ± 3.8	4998.3 ± 7.8	2043.3 ± 5.3	3717.4 ± 8.4	1635.4 ± 10.6
**227**	2953 ± 10.5	1546.2 ± 7.2	3229.7 ± 1.4	1383 ± 5.6	5014.9 ± 3.3	2148.5 ± 9.1	3846.5 ± 8.9	1724.4 ± 5.9
**279**	2900.8 ± 9.8	1612.3 ± 8	3450.4 ± 14	1448.2 ± 1.8	5010.6 ± 1.6	2260.5 ± 8.8	4006.8 ± 13.2	1813.9 ± 9.7
**341**	2772.1 ± 4.8	1709.2 ± 3.6	3734.7 ± **101**	1520.9 ± 2.2	4929 ± 3.6	2372 ± 5.3	4174.8 ± 8.9	1914.4 ± 8.4
**418**	2490.3 ± 7.4	1761.2 ± 8.6	4094.9 ± 3.7	1581.2 ± 6.1	4722.3 ± 11.2	2482.3 ± 3.7	4374.3 ± 9.6	2006.9 ± 4.6
**513**	1953.6 ± 7.9	1826.9 ± 3.5	4538.6 ± 2.1	1661.2 ± 5.9	4293.4 ± 6.6	2586 ± 4.7	4532.2 ± 5.7	2113.5 ± 3.2
**628**	1418.6 ± 8.8	1873.7 ± 3.1	5362.5 ± 5.5	1814.6 ± 6.7	3707.2 ± 11	2818.3 ± 9.7	4936.2 ± 8.7	2338.4 ± 2.4

Results (displayed as mean values ±SD, n = 3), SM – storage modulus, LM – loss modulus.
